# Socioeconomic Deprivation in Adolescents Hospitalized with Anorexia Nervosa and Atypical Anorexia Nervosa

**DOI:** 10.3390/children13060808

**Published:** 2026-06-12

**Authors:** Anita V. Chaphekar, James Peugh, Andrew F. Beck, Carolina M. Bejarano, Danielle Scharf, Kimberly Stevens, Claire M. Aarnio-Peterson

**Affiliations:** 1 Division of Adolescent and Young Adult Medicine, Cincinnati Children’s Hospital Medical Center, Cincinnati, OH 45229, USA; 2 Department of Pediatrics, University of Cincinnati College of Medicine, Cincinnati, OH 45229, USA; 3 Division of Behavioral Medicine and Clinical Psychology, Cincinnati Children’s Hospital Medical Center, Cincinnati, OH 45229, USA; 4 Division of General and Community Pediatrics, Cincinnati Children’s Hospital Medical Center, Cincinnati, OH 45229, USA; 5 Division of Hospital Medicine, Cincinnati Children’s Hospital Medical Center, Cincinnati, OH 45229, USA

**Keywords:** anorexia nervosa, atypical anorexia nervosa, eating disorder, inpatient, deprivation, area deprivation index

## Abstract

**Highlights:**

**What are the main findings?**

- More deprived areas were associated with lower percentage of target goal weight achieved at 3-month follow up.
- More deprived areas were associated with shorter length of hospitalization.

**What is the implication of the main finding?**

- This study adds a measure of socioeconomic deprivation, the deprivation index, which has not previously been studied in individuals hospitalized for eating disorders.

**Abstract:**

**Objectives:** We sought to examine associations between area-level socioeconomic deprivation and: (1) hospitalization length; (2) severity; and (3) clinical outcomes in adolescents hospitalized for atypical anorexia nervosa or anorexia nervosa (AN). We hypothesized that more socioeconomic deprivation would be associated with longer length of hospitalization, greater illness severity, and poorer clinical outcomes. **Methods:** We conducted a study of individuals aged 11–18 years with AN who were admitted for medical instability. Tract-level socioeconomic deprivation was characterized by using a deprivation index (range 0–1, higher scores indicating more deprivation). Outcomes of interest included (1) hospitalization length defined as duration in days between admission and discharge, (2) illness severity defined as percentage of goal weight at admission and global Eating Disorder Examination Questionnaire (EDE-Q) score and (3) clinical outcomes defined as re-admission rate, 3- and 6-month follow up percentage goal weight, and 3- and 6-month weight restoration status. We used multivariate regression to assess associations between deprivation index and outcomes of interest. **Results:** A total of 121 adolescents were included. The mean age was 15.4 years (range 11.0–17.9 years). Higher deprivation was associated with a lower percent of target goal weight achieved at 3-month follow up (Estimate= −19.8; *p*-value = 0.029; 95% CI= −37.5 to −2.1). Contrary to our initial hypothesis, higher deprivation index scores were associated with shorter hospitalization lengths (Estimate = −15.4; *p* < 0.001; 95% CI = −22.6 to −8.2). **Conclusions:** Higher deprivation was associated with a lower percent of target goal weight achieved at 3-month follow up and shorter length of hospitalization. This study adds to the extant literature by using a novel method, geocoding, to examine the relationship between socioeconomic deprivation and eating disorder outcomes. Future studies with more diverse samples (including Spanish speakers) are needed.

## 1. Introduction

Eating disorders, such as anorexia nervosa or atypical anorexia nervosa (both referred to as AN from this point forward), can have serious medical complications and increased burden on quality of life [1,2]. Eating disorder rates among adolescents have increased in recent years with corresponding rising numbers of inpatient hospitalizations since 2020 [3]. Given this context, it is important to understand the factors that influence eating disorder presentation, severity, and outcomes.

Bronfenbrenner’s Ecological Systems Theory suggests an individual’s development is influenced by interconnecting ecological systems such as one’s family to broader structural factors such as one’s neighborhood environment [4]. This theory provides the framework that health outcomes are influenced by social determinants of health, defined “as the conditions in which people are born, grow, live, work and age, and people’s access to power, money and resources” [5]. This is evident in the pediatric population as socioeconomic disparities are linked to worsened health outcomes, such as longer and more frequent inpatient stays, and increased exacerbations across chronic conditions [6,7]. However, there is a dearth of research examining social determinants of health in the eating disorder population [8].

Prior studies examining the relationship between socioeconomic deprivation and eating disorders have shown mixed results. Few studies examining socioeconomic deprivation separate eating disorders into specific diagnoses, representing a gap in the current literature; thus, we will describe the previous research for eating disorders in general. Evidence suggests that individuals with parents who have a higher income, education status, or live in wealthier areas, have an increased incidence of eating disorders [9,10,11,12,13,14,15,16,17]. However, other studies have shown the opposite to be true, that individuals in more deprived areas are at an increased risk of developing an eating disorder and having worsened eating disorder symptoms [9,18,19,20,21,22,23,24,25,26,27]. These mixed findings highlight key gaps in our understanding of how social determinants of health influence eating disorder severity and outcomes. Moreover, much of the existing research has been conducted with predominantly higher-income, well-resourced populations, limiting generalizability to youth living in more deprived areas and those of lower socioeconomic status [28]. Additionally, referral and diagnostic biases may contribute to the overrepresentation of individuals with higher socioeconomic status in clinical samples, further obscuring the true relationship between socioeconomic factors and eating disorder outcomes.

Area-level deprivation indices can be useful markers for an individual’s socioeconomic environment and population-level risk factors [29,30,31,32,33]. Furthermore, deprivation indices can provide a more nuanced measure of socioeconomic disparities compared to income or insurance status alone, illustrating key characteristics of the places where individuals live [34]. The purpose of this study was to determine associations between area-level socioeconomic deprivation, as defined by a deprivation index score (derived from a geocoded home address), and length of hospitalization, illness severity (percentage of goal weight at admission and the Eating Disorder Examination Questionnaire, or EDE-Q, global score), and clinical outcomes (percentage of goal weight at 3 and 6 months, weight restoration status, and readmission rate) in individuals treated for an eating disorder who were initially hospitalized. To the best of our knowledge, this novel method has not been previously used in inpatient eating disorder samples. We hypothesized that higher socioeconomic deprivation will be associated with longer hospitalization, greater illness severity, and poorer clinical outcomes.

## 2. Materials and Methods

### 2.1. Study Design and Data Source

This was a cohort study of 121 individuals aged 11 to 18 years with AN who were admitted for medical instability at a large pediatric medical center in the Midwestern United States between July 2017 and January 2019. As anorexia nervosa and atypical anorexia nervosa have been shown to have similar clinical presentations and illness severity, individuals with either diagnosis were classified as ‘AN’ [35,36]. Medical instability was defined by the Society for Adolescent Health and Medicine medical management of restrictive eating disorders and included vital signs such as heart rate lower than 50 beats per minute and/or electrolyte imbalances [37]. All patients with AN admitted to the inpatient Eating Disorders Program were asked to complete clinical questionnaires upon admission as part of routine clinical care to inform treatment. A member of the research team approached each eligible patient to obtain informed consent to be able to use their de-identified data for research purposes. Only data from patients who gave informed consent were included in the research study. Exclusion criteria for this study included caregivers that did not speak fluent English and participants with prior eating disorder treatment. Caregivers that did not speak fluent English were excluded as the questionnaires for this study were not translated to another language at the time of the original study. Participants with prior eating disorder treatment were excluded to maintain consistency with the larger parent study eligibility criteria. This study was approved by the Institutional Review Board #2017-2569 at Cincinnati Children’s Hospital Medical Center.

### 2.2. Outcome Variables

Outcomes of interest focused on index hospitalization characteristics, illness severity, and a variety of clinical measures. Length of the initial hospitalization was defined as the days from the first date of admission to the date of discharge. We defined illness severity as the percentage of goal weight at admission and the Eating Disorder Examination Questionnaire (EDE-Q) global score. Goal weight was determined using an individual’s developmental growth trends (rather than using median body mass index given roughly half of the sample was diagnosed with atypical anorexia nervosa). Goal weight was calculated by inputting each participant’s height into their individualized, expected body mass index percentile for age and sex. Percentage goal weight was determined by dividing the participant’s actual weight by their goal weight and multiplying by 100. Eating disorder symptom severity was determined with the EDE-Q, collected as part of the clinical interview with the participant [38]. The EDE-Q is a 28-item self-report assessment of eating disorder symptoms over the last 28 days. From the EDE-Q, a global score and 4 subscale scores of restraint, shape concern, weight concern, and eating concern are generated. The EDE-Q global score ranges from 0 to 6. The global scores were used for this study. Higher EDE-Q scores indicate greater symptom severity [39].

We also assessed several clinical outcome measures. These included percentage of goal weight at 3 and 6 months and weight restoration status at 3 and 6 months. Weights at 3- and 6-month post-hospital discharge clinic visits were obtained from chart review and percentage of goal weight at 3 and 6 months was determined as described above. Weight restoration status was defined as a participant reaching (at or above 95% of) their goal weight at 6 months. Additionally, chart review was utilized to determine if a participant was readmitted to the hospital within 6 months of initial hospitalization.

### 2.3. Exposure Variable and Covariates

The exposure of interest was an area-level measure of deprivation, known as the Community Material Deprivation Index, measured at the census tract level [40]. Patient street addresses were obtained from the electronic health record and were then geocoded using DeGAUSS software [41]. Once geocoded, addresses were spatially joined to census tract geographies. This enabled linkage to census tract-level deprivation index scores [42,43]. The deprivation index ranges from 0 to 1. A value closer to 0 correlates with a less deprived area and values closer to 1 correlate with a more deprived area. This score was calculated using pre-existing variables from the 2015 US Census American Community Survey. The 6 variables composing this index are: fraction of households below the federal poverty level, median annual household income, fraction of adults with less than high school education, fraction of population without health insurance, fraction of households receiving public assistance, and fraction of housing units that are vacant [41].

We extracted several additional demographic and clinical variables from the electronic health record as covariates. These included age, sex (male or female), race (White or other), ethnicity (Hispanic or Non-Hispanic), insurance type (public insurance or private insurance), and diagnosis (anorexia nervosa or atypical anorexia nervosa).

### 2.4. Statistical Analyses

Deprivation index score was examined as a continuous variable. We used multiple linear regression to assess associations between continuous outcome variables and logistic regression for binary outcomes (weight restoration). Statistical analyses were conducted using Mplus (Version 8.11) [42].

## 3. Results

A total of 121 patients met inclusion criteria. The average age of individuals in this study was 15.4 years (range 11.0–17.9 years, SD = 1.5 years). Females accounted for 81.8% of the study population. Additionally, the majority identified as White (96.7%) and Non-Hispanic (97.5%); similarly, most had private insurance (88.5%). A total of 52.9% of patients were diagnosed with anorexia nervosa and 47.1% with atypical anorexia nervosa. The mean deprivation index score of this sample was 0.3 (range 0.1 to 0.6, SD = 0.1). See Table 1 for additional sample characteristics.

Higher deprivation was associated with a lower percent of target goal weight achieved at 3-month follow up (Estimate= −19.8; *p*-value = 0.029; 95% CI= −37.5 to −2.1) and lower odds of weight restoration at 3-month follow up (Estimate= −5.7; *p*-value = 0.048; 95% CI = −11.3 to −0.1). A higher deprivation index score was associated with a shorter length of hospitalization (Estimate = −15.4; *p* < 0.001; 95% CI = −22.6 to −8.2). There were no significant differences based on deprivation scores for the remaining illness severity and clinical outcomes (Table 2).

## 4. Discussion

To the best of our knowledge, this is the first study to examine socioeconomic deprivation using a novel methodology, geocoding, in the inpatient eating disorder population. The mean deprivation index score of our population was similar to the mean deprivation index score of other research studies conducted in the region [29]. Counter to our initial hypothesis, we found that more deprivation was associated with a shorter length of hospitalization. This finding is similar to a recently published study that showed a correlation between higher household income and longer length of stay among patients with eating disorders [43]. It is important to consider possible explanations of the relationship between deprivation and length of hospitalization. One possible explanation is individuals and families residing in less deprived areas have less financial constraint with insurance or medical coverage allowing for longer hospitalization. Additionally, individuals in less deprived areas could have increased health literacy and thus advocate for a longer hospital stay [44]. However, given the limited diversity of our sample, we recommend interpreting these findings with extreme caution.

Higher deprivation was associated with both lower percentage of target goal weight achieved and weight restoration status at 3-month follow-up which is consistent with our initial hypothesis. While the mechanism of this relationship was beyond the scope of this study, this association likely reflects the interaction between various social and environmental factors. Factors such as food insecurity, barriers to care, and increased overall stress may all contribute to this observed relationship between deprivation and percentage of goal weight achieved at 3-month follow-up. Specific mechanisms of contributing factors should be the focus of future studies.

Counter to our initial hypothesis, there were no significant differences found between deprivation index and other measured illness severity or clinical outcomes. However, these null findings should be interpreted with great caution given the limited diversity in our sample. There is very limited heterogeneity of race, ethnicity, and higher deprivation areas as the majority of individuals in this study identified as White, Non-Hispanic, and living in less-deprived areas. Accordingly, the absence of statistically significant findings should not be construed as evidence of no association between deprivation and eating disorder outcomes. Rather, these results underscore the need for studies with diverse samples to more rigorously evaluate this relationship. At the same time, our findings demonstrate the novelty and feasibility of employing an area-level deprivation index to capture socioeconomic context beyond individual-level social determinants, [21,45,46] thereby offering a preliminary framework to inform future research.

It is also important to keep in mind the existing nuances in relationships between socioeconomic factors and eating disorders. Larson et al. (2021) found associations between socioeconomic status and disordered eating differed by gender and were no longer significant after adjusting for body mass index (BMI) and ethnicity [21]. Larson’s findings suggest an intersecting and multifaceted relationship between socioeconomic factors and eating disorders. These nuances could help explain why, within our cohort where severity in eating disorder illness was high (cohort was all medically hospitalized) and where there was a lack of racial and ethnic diversity, we did not observe clear relationships between deprivation and illness severity or clinical outcomes.

There are several limitations to this study. Our sample size was small and thus the ability to make comparisons across levels of deprivation was limited. Also, most of our sample identified as White and Non-Hispanic, and had private insurance, thereby further limiting the generalizability of our findings. Barriers to care and biases influencing diagnosis could contribute to the lack of representativeness in our sample. Additionally, the language exclusion criteria for this study (and the parent study) reduced representation of diverse families. An additional limitation of this study lies in the distribution of deprivation index values. While the mean deprivation index score was comparable with other studies done in our region [29], this may not generalize to other regions. Moreover, the distribution of deprivation in our study was left-skewed with far more patients clustered in lower deprivation tracts. Another limitation of our study was that we did not account for the potential of address changes in our cohort, which could have resulted in misclassification of deprivation index. Finally, although previous studies have shown correlations between area- and individual-level socioeconomic status [33], our exposure of area-level deprivation should be interpreted as a marker of one’s context and not as a true marker of individual-level socioeconomic deprivation [47].

## 5. Conclusions

This study lays the groundwork for future studies with greater socioeconomic and racial/ethnic diversity. The use of deprivation indices represents a novel approach for contextualizing socioeconomic factors in this population. Such data could facilitate identification of risk factors not traditionally captured during hospital stays by individual social factors such as income. Future studies should include samples with greater diversity to leverage the use of deprivation indices to better understand how socioeconomic context may shape eating disorder trajectories.

## Figures and Tables

**Table 1:** Demographic and clinical characteristics among adolescents with anorexia nervosa or atypical anorexia nervosa admitted for medical stabilization.

	Overall Cohort, N = 121
**Age (mean ± SD)**	15.4 ± 1.5
**Race, N (%)**	
**White**	117 (96.7)
**Other**	4 (3.3)
**Ethnicity, N (%)**	
**Non-Hispanic**	118 (97.5)
**Other**	2 (1.7)
**Gender, N (%)**	
**Female**	99 (81.8)
**Male**	22 (18.2)
**Insurance, N (%)**	
**Public**	13 (11.5)
**Private**	100 (88.5)
**Deprivation index (mean ± SD)**	0.3 ± 0.1
**Diagnosis**	
**Anorexia nervosa, N (%)**	64 (52.9)
**Atypical anorexia nervosa, N (%)**	57 (47.1)
**Percent goal weight (mean ± SD)**	76.6 ± 8.8
**EDE-Q score (mean ± SD)**	3.2 ± 1.7
**Percent goal weight achieved at 3 months (mean ± SD)**	87.5 ± 9.4
**Percent goal weight achieved at 6 months (mean ± SD)**	88.6 ± 9.1
**Weight restoration status at 3 months, N (%)**	29 (24.0)
**Weight restoration status at 6 months, N (%)**	26 (21.5)

**Table 2:** Associations between deprivation index.

	Deprivation Index		
	Estimate	95% Confidence Interval	*p*-Value
**Length of hospitalization**	**−15.370**	**−22.589 to −8.151**	**<0.001**
**Illness severity**			
**Percent goal weight**	−2.714	−19.844 to 14.416	0.756
**Global EDE-Q score**	1.714	−1.187 to 4.615	0.247
**Clinical outcomes**			
**Percent goal weight achieved at 3 months**	**−19.778**	**−37.496 to −2.060**	**0.029**
**Percent goal weight achieved at 6 months**	−12.913	−28.050 to 2.224	0.095
**Weight restoration status at 3 months**	**−5.662**	**−11.274 to −0.051**	**0.048**
**Weight restoration status at 6 months**	−0.019	−4.871 to 4.883	0.994

Bold formatting indicates significant *p*-values.

## Data Availability

The datasets generated and/or analyzed during the current study are available from the corresponding author on reasonable request. The data are not publicly available due to privacy or ethical restrictions.
